# The Effect of Sex and Stature on Footprint Patterns Among Students of Different Age Groups in a South Indian Medical College

**DOI:** 10.7759/cureus.97711

**Published:** 2025-11-24

**Authors:** Yogesh C, Spandana J Kabbur, Rakshatha Naik, K Sudheer

**Affiliations:** 1 Forensic Medicine, Subbaiah Institute of Medical Sciences and Research Centre, Shivamogga, IND; 2 Preventive Medicine, Subbaiah Institute of Medical Sciences and Research Centre, Shivamogga, IND

**Keywords:** anthropometry, correlation, forensic anthropology, sex determination, south indian population

## Abstract

Sex and stature determination from footprints found at crime scenes can narrow down possibilities and help forensic investigation. Personal identification is very useful and plays a crucial role in situations where skeletal remains are not available. Among all biological parameters present, stature serves as an important identifying characteristic for narrowing down the possibilities of an unknown individual. Among all evidence, footprints are most commonly seen in crime scenes and disaster sites. Being easily obtainable, these provide valuable information in finding the biological profile of an individual. This study aims to find a possible correlation between footprint measurements and stature in a sample of 200 right footprints collected from 100 men and 100 women ranging from 18 to 23 years in age. This study also aims to find sexual dimorphism between footprint dimensions and the stature of an individual. Seven measurements were taken from each footprint, which include five lengths (T1 to T5) and two breadths (ball breadth (BB) and heel breadth (BH)) to correlate with the stature of an individual. The stature of individuals was also measured using a stadiometer. Pearson's correlation coefficient (r) was calculated for each length with stature to find which footprint dimension correlates most with the stature of an individual. Student's independent t-test was also applied to compare the footprint dimensions and stature between male and female participants.

When both sexes were analyzed together, positive correlation with stature was observed in all measurements, with ball breadth showing the highest association (r=0.473, p<0.001). An independent t-test showed statistically significant differences (p<0.05) in all footprint dimensions and stature between male and female participants, with higher values consistently observed in men. The study concludes that footprint dimensions can serve as a dependable tool for distinguishing sex and estimating stature. All recorded footprint parameters displayed clear sexual dimorphism. While the correlations with stature were moderate, they were statistically significant, highlighting the usefulness of footprints in forensic identification.

## Introduction

A footprint is a static impression of the plantar surface of the human foot produced when the sole comes into contact with the floor. It represents a unique morphological record mostly used in forensic and anthropometric sciences owing to its strong association with individual identification. A footprint may be normal, flat, curved, or any of the infinite variations between the main types. Pointed toes, flat toes, wide heel, narrow heel, and many intermediate variations are seen in the present population. Sex, stature, and age are the basic characteristics required for personal identification. Similarly, fingerprints, footprints, lip prints, palm prints, and ear prints are used in forensic investigations to identify a person through comparative analysis [1]. Many techniques are available for determining age, sex, stature, and race, but this process is complicated by differences in children's growth rates.

Hence, this study only aims at identification using static adult footprints [2]. The study addresses the following gap.

Region-specific data

Anthropometric correlations are known to be geographically specific. Our study provides necessary, contemporary, and systematic data on distinct footprint dimensions from a defined South Indian cohort.

Foundation for models

By confirming significant sexual dimorphism and moderate correlation, this research provides essential descriptive data needed for the subsequent development of accurate, sex-specific regression equations for stature estimation. Although multiple studies have examined footprint-based identification, its application in routine forensic identification remains less explored compared to skeletal and dental parameters. Personal identification is defined as determining a person's individuality. It also refers to the process of linking an unknown person, personal object, or any material back to a person of known identity [3]. Stature is difficult to estimate in adolescents because of the ongoing physical growth of the thorax region and the long bones of the lower limbs, which substantially contribute to the stature of an individual. Past studies on stature estimation have been largely conducted on the adult population, primarily due to the adolescent growth spurt and the effect of growth on the long bones of the body [4]. The principal characteristics for identifying an individual are age, sex, stature, and race. In cases where other evidence is not available, footprints can still be collected and used to identify a person [5].

The structure of the human foot, like the rest of the skeleton, is shaped by its mechanical environment during growth and development of an individual. The morphology of the foot reflects the habitual load and movement patterns that it adapts throughout life. In particular, footprint dimensions mirror the structural adaptations of the foot that support locomotion. Thus, these measurements provide valuable insight into anatomical variations arising from sex-based differences. Although numerous other parameters, such as the femur and humerus, have been used mostly in stature determination, footprint-based estimation plays a major role when these parameters are not available. Moreover, footprint-based analysis is not widely explored in identification studies. Footprints exhibit variations; thus, this study aims to evaluate differences and their role in estimating stature and sex using these measurements [6].

Building an individual's biological profile by assessing sex, age, and stature is the most vital step in personal identification, which helps in identifying unknown decomposed bodies and skeletal remains. Sex identification is a key factor in establishing an individual's identity because later methods of estimating sex and stature rely on it [7]. Determining sex reduces the number of possible identities [8]. One of the primary objectives of forensic anthropology is the determination of sex by human remains, whether skeletal, decomposed, mutilated, or even cremated. In forensic osteology, sex identification plays a crucial role and depends on the application of updated analytical methods. Similarly, footprints can serve as valuable indicators for sex estimation when complete skeletal remains are not available, providing an alternative yet effective approach in forensic investigations [9].

A footprint is a mark made by the weight-bearing parts of the sole of the foot. They may appear on rainwater-soaked ground, freshly polished floors, fresh cement, damp surface, or dust, mud, sand, oil, paint, and even in blood in crime scenes [10]. In such scenes, footprints are often found because the accused often eliminate their footwear to avoid sound and to have a firm grip while climbing walls or moving on the floor. Like fingerprints, footprints are also unique for a person. Therefore, footprints found can be matched with the footprints of suspects to confirm or exclude them from involvement in the crime [11]. Criminals nowadays use various methods to avoid leaving behind their fingerprints, such as wearing gloves and wiping with wet clothes. However, masking these footprints is still uncommon among these offenders, which may play an important role in catching them [12].

This study was conducted to determine the correlation between the seven dimensions of footprints, which involves five lengths (T1 to T5), which are the measurements taken from each toe to the posterior midpoint of the heel, and two breadths, which include ball breadth (BB) and heel breadth (BH), and the stature and sex of an individual in South Indian adults, particularly considering the changes in built and dimensions of the footprints based on various geographical locations and ethnicities of human beings. Although several international and North Indian studies have developed regression models for stature estimation from footprints, there is a lack of population-specific data and regression equations for South Indian adults, creating a significant gap that this study aims to address.

The primary objective of the study is to determine the correlation between footprint dimensions and stature. The secondary objective is to assess sexual dimorphism in footprint parameters.

## Materials and methods

This study was conducted in a private medical college in Shivamogga, Karnataka, India, after obtaining Institutional Ethics Committee approval (reference number: IEC-SUIMS/61/2025-26). Participants' consent was obtained, and all collected data were anonymized to ensure privacy and confidentiality. The minimum required sample size was calculated using the following formula: n = [(Zα + Zβ)/C]^2 + 3, where C = 0.5 × ln[(1 + r)/(1 − r)], assuming r = 0.36, α = 0.05, and power = 80%, yielding approximately 60 participants per sex. The sample size was increased to 100 per sex for higher reliability [4]. To study these inked barefoot prints, 200 participants of South Indian origin were selected [6]. All participants were students of medical, dental, and nursing programs from a single medical college. However, there are students studying in the institute who are not of South Indian origin. Hence, the phrase "South Indian origin" says that the participants chosen were from South Indian origin only. The study was conducted in the Department of Forensic Medicine and Toxicology. After obtaining consent, the participants were grouped separately according to their age group and sex. Stratified systematic sampling was applied, dividing the sample based on sex and age groups. Participants were randomly selected from students of medical, dental, and nursing programs to minimize selection bias. The study included participants distributed across five age groups: 18 years to 18 years 364 days, 19 years to 19 years 364 days, 20 years to 20 years 364 days, 21 years to 21 years 364 days, and 22 years to 22 years 364 days. Each age group comprised 20 male and 20 female participants.

We used a stratified systematic sampling method to ensure equal representation across both sexes and different age groups. The population was first divided into strata based on sex and age. Within each stratum, participants were selected among first, second, third, and fourth year students of all medical, dental, and nursing courses to maintain diversity while ensuring fair distribution.

**Figure 1:**
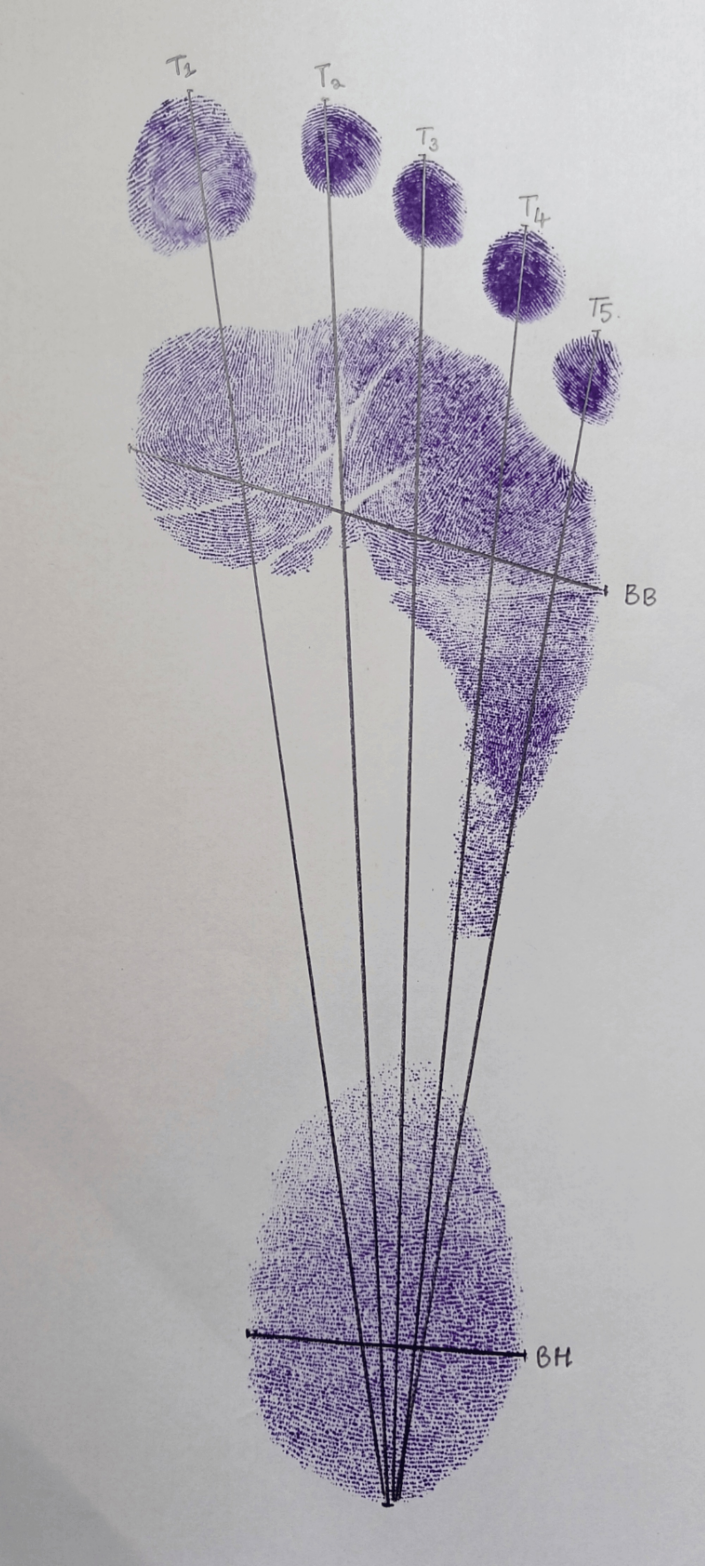
Landmarks and measurements on footprint T1: distance from the great toe to the most posterior point of the heel, T2: distance from the second toe to the most posterior point of the heel, T3: distance from the third toe to the most posterior point of the heel, T4: distance from the fourth toe to the most posterior point of the heel, T5: distance from the fifth toe to the most posterior point of the heel, BB: the maximum horizontal distance across the ball of the foot, measured between the most medial point of the head of the first metatarsal and most lateral point of the head of the fifth metatarsal, BH: the maximum horizontal distance across the heel, measured between the most medial and lateral points of the heel

A total of 200 right footprints were obtained from 100 male and 100 female participants. Subjects with foot deformities, injuries, or orthopedic conditions were excluded. Proper consent was taken on the consent form explaining the procedure to the respective participants. All measurements were taken between 9 AM and 12 PM to minimize diurnal variation in stature. Only the right foot was analyzed for uniformity, and participants with foot deformities or injuries were excluded to avoid confounding factors. While taking the footprints, precautions were taken, such as making the subjects clean their soles with soap and water before taking the sample. Then, a thin and uniform layer of ink was applied to the soles of the subjects, and they were asked to step on a white paper placed on a flat surface. The obtained footprint was allowed to dry and used for measurements. Only the right footprint was analyzed to maintain uniformity across the dataset and minimize variability from side dominance (Figure 1).

The subjects' stature was also measured according to standard procedures as recommended by previous studies, along with footprints [9]. The stadiometer was placed on a flat surface to ensure that the vertical column was perpendicular to the ground. The subjects were made to remove any footwear, caps, etc., that could alter height. Subjects were made to stand barefoot on the stadiometer baseboard with their heels together, touching the vertical board, with their legs straight, arms at their sides, and shoulders relaxed. Occipital protuberance, shoulder blades, buttocks, calves, and heels were touching the vertical board of the stadiometer. The Frankfort horizontal plane was used for head positioning; i.e., the horizontal line joining the lowest point of the eye socket to the upper border of the ear canal was parallel to the floor.

During analysis, seven measurements were recorded using a Vernier caliper from each footprint collected, comprising five lengths and two breadths. Maximum care was taken to avoid errors due to intra-observer variations. The following footprint length measurements were taken: T1, distance from the great toe to the most posterior point of the heel; T2, distance from the second toe to the most posterior point of the heel; T3, distance from the third toe to the most posterior point of the heel; T4, distance from the fourth toe to the most posterior point of the heel; T5, distance from the fifth toe to the most posterior point of the heel; BB, the maximum horizontal distance across the ball of the foot, measured between the most medial point of the head of the first metatarsal and the most lateral point of the head of the fifth metatarsal; and BH, the maximum horizontal distance across the heel, measured between the most medial and lateral points of the heel. The following standardized anthropometric landmarks were used: the most anterior point of each toe tip (T1-T5) and the most posterior point of the heel for length measurements; the most medial point of the head of the first metatarsal and the most lateral point of the head of the fifth metatarsal for ball breadth (BB); and the most medial and lateral points of the heel for heel breadth (BH). All landmarks were identified visually and measured using a Vernier caliper to ensure consistency.

The obtained data were compiled and analyzed using the SPSS software (IBM Corp., Armonk, NY) with the assistance of a qualified biostatistician. The distribution of the measurements was checked for normality.

Based on previous studies, the minimum required sample size for detecting the correlation coefficient (r) was calculated to be 0.36 at a 95% confidence limit and 80% power, which was approximately 60 participants from each sex. However, to improve the reliability and accuracy of the study, a total of 100 participants were selected from both male and female sexes for analysis [11].

## Results

The means, standard deviations, and ranges of footprint measurements of all the subjects are shown in Table 1. The different variations in the measurements of variables can be seen by looking at the minimum and maximum columns of Table 1.

**Table 1:** Description statistics of footprint measurements in both male and female populations combined

Measurements	Number	Minimum	Maximum	Mean	Standard deviation
	Statistic	Statistic	Statistic	Statistic	Statistic
T1 (cm)	200	18.70	28.60	23.67	1.99
T2 (cm)	200	18.40	28.30	23.59	2.11
T3 (cm)	200	17.70	27.80	22.82	1.98
T4 (cm)	200	17.10	25.80	21.56	1.81
T5 (cm)	200	16.00	25.60	20.06	1.70
BB (cm)	200	4.30	11.40	8.88	1.10
BH (cm)	200	0.60	7.50	5.03	0.86
Height (cm)	200	145.00	193.00	165.74	10.57
Valid number (listwise)	200	-	-	-	-

Normality of data was confirmed using the Shapiro-Wilk test (p>0.05). All measurements were taken by a single trained observer to minimize intra-observer error. Regression equations were formulated for the most significant footprint parameters to aid stature estimation. The footprint dimensions that were subjected to statistical analysis to find Pearson's correlation coefficient (r) with stature showed moderate positive correlation with stature, with r-values ranging from 0.340 to 0.473; these correlations were statistically significant with a p-value of <0.05. The reported r-value represents the overall correlation for both sexes combined, rather than separately for men and women. Among all the footprint measurements, the highest correlation with stature was observed for ball breadth (BB), as seen in Table 2.

**Table 2:** Pearson's correlation coefficient (r) values between footprint measurements and stature in both sexes **Correlation is significant at the 0.01 level (two-tailed).

Measurements		T1 (cm)	T2 (cm)	T3 (cm)	T4 (cm)	T5 (cm)	BB (cm)	BH (cm)	Height (cm)
T1 (cm)	Pearson correlation (r)	1	0.789^**^	0.671^**^	0.630^**^	0.521^**^	0.460^**^	0.451^**^	0.377^**^
	Sig. (2-tailed)	-	0.000	0.000	0.000	0.000	0.000	0.000	0.000
	Number	200	200	200	200	200	200	200	200
T2 (cm)	Pearson correlation (r)	0.789^**^	1	0.670^**^	0.623^**^	0.570^**^	0.405^**^	0.340^**^	0.340^**^
	Sig. (2-tailed)	0.000	-	0.000	0.000	0.000	0.000	0.000	0.000
	Number	200	200	200	200	200	200	200	200
T3 (cm)	Pearson correlation (r)	0.671^**^	0.670^**^	1	0.832^**^	0.693^**^	0.628^**^	0.580^**^	0.461^**^
	Sig. (2-tailed)	0.000	0.000	-	0.000	0.000	0.000	0.000	0.000
	Number	200	200	200	200	200	200	200	200
T4 (cm)	Pearson correlation (r)	0.630^**^	0.623^**^	0.832^**^	1	0.745^**^	0.616^**^	0.608^**^	0.467^**^
	Sig. (2-tailed)	0.000	0.000	0.000	-	0.000	0.000	0.000	0.000
	Number	200	200	200	200	200	200	200	200
T5 (cm)	Pearson correlation (r)	0.521^**^	0.570^**^	0.693^**^	0.745^**^	1	0.604^**^	0.547^**^	0.467^**^
	Sig. (2-tailed)	0.000	0.000	0.000	0.000	-	0.000	0.000	0.000
	Number	200	200	200	200	200	200	200	200
BB (cm)	Pearson correlation (r)	0.460^**^	0.405^**^	0.628^**^	0.616^**^	0.604^**^	1	0.728^**^	0.473^**^
	Sig. (2-tailed)	0.000	0.000	0.000	0.000	0.000	-	0.000	0.000
	Number	200	200	200	200	200	200	200	200
BH (cm)	Pearson correlation (r)	0.451^**^	0.340^**^	0.580^**^	0.608^**^	0.547^**^	0.728^**^	1	0.411^**^
	Sig. (2-tailed)	0.000	0.000	0.000	0.000	0.000	0.000	-	0.000
	Number	200	200	200	200	200	200	200	200
Height (cm)	Pearson correlation (r)	0.377^**^	0.340^**^	0.461^**^	0.467^**^	0.467^**^	0.473^**^	0.411^**^	1
	Sig. (2-tailed)	0.000	0.000	0.000	0.000	0.000	0.000	0.000	-
	Number	200	200	200	200	200	200	200	200

An independent Student's t-test was applied to compare the footprint measurements and stature between male and female participants. Men consistently demonstrated higher mean values across all parameters compared with women. The results revealed statistically significant differences across all parameters. For lengths, male participants had significantly greater values than female participants, with t(198)=-13.87 for T1, t(198)=-9.28 for T2, t(198)=-10.92 for T3, t(198)=-10.26 for T4, and t(198)=-8.76 for T5, with a significant p-value of <0.001. Similar trends were observed for the breadth dimensions, where male participants showed higher values compared to female participants, with t(198)=-13.46 for BB and t(198)=-9.18 for BH, with a p-value of <0.001. Stature also showed a significant sex difference, with men being taller than women (t(198)=-8.15, p<0.001). Overall, the analysis shown in Table 3 justifies that all measured footprints were significantly greater in male participants than in female participants.

**Table 3:** Value of "t" for the sexual difference between male and female participants' footprint measurements Sig. (2-tailed) is the p-value. Mean difference indicates the male-female difference in cm. Confidence intervals are at the 95% level.

Measurements		Levene's test for equality of variances		t-test for equality of means						
		F	Significance	t	df	Sig. (2-tailed)	Mean difference	Standard error of the difference	95% confidence interval of the difference	
									Lower	Upper
T1 (cm)	Equal variances assumed	6.636	0.011	-13.866	198	0.000	-2.79090	0.20128	-3.18782	-2.39398
	Equal variances not assumed	-	-	-13.866	186.609	0.000	-2.79090	0.20128	-3.18797	-2.39383
T2 (cm)	Equal variances assumed	3.194	0.075	-9.280	198	0.000	-2.3190000	0.2498867	########	-1.8262191
	Equal variances not assumed	-	-	-9.280	188.437	0.000	-2.3190000	0.2498867	########	-1.8260653
T3 (cm)	Equal variances assumed	1.529	0.218	-10.924	198	0.000	-2.4205000	0.2215724	########	-1.9835553
	Equal variances not assumed	-	-	-10.924	195.432	0.000	-2.4205000	0.2215724	########	-1.9835200
T4 (cm)	Equal variances assumed	3.131	0.078	-10.258	198	0.000	-2.1255000	0.2072125	########	-1.7168733
	Equal variances not assumed	-	-	-10.258	190.862	0.000	-2.1255000	0.2072125	########	-1.7167794
T5 (cm)	Equal variances assumed	0.275	0.600	-8.763	198	0.000	-1.7975000	0.2051131	########	-1.3930134
	Equal variances not assumed	-	-	-8.763	196.884	0.000	-1.7975000	0.2051131	########	-1.3929993
BB (cm)	Equal variances assumed	9.887	0.002	-13.455	198	0.000	-1.5111000	0.1123048	########	-1.2896330
	Equal variances not assumed	-	-	-13.455	190.265	0.000	-1.5111000	0.1123048	########	-1.2895777
BH (cm)	Equal variances assumed	17.759	0.000	-9.178	198	0.000	-0.9355000	0.1019329	########	-0.7344865
	Equal variances not assumed	-	-	-9.178	182.061	0.000	-0.9355000	0.1019329	########	-0.7343782
Height (cm)	Equal variances assumed	6.753	0.010	-8.150	198	0.000	-10.570	1.297	-13.128	-8.012
	Equal variances not assumed	-	-	-8.150	187.649	0.000	-10.570	1.297	-13.128	-8.012

## Discussion

The significance of determining sex and stature in personal identification and analyzing various prints at crime scenes is well recognized [13]. Although it is a key element in creating a personal biological profile, research on sex estimation from footprints is limited [14]. Human stature and build are strongly correlated with body measurements, which are often used in metric studies for sex determination. Stature and build are also crucial for personal identification in forensic investigation, and therefore, many studies have relied on morpho-geometric measures of various body parts to estimate height. Previous researchers have also studied the foot for its usefulness in height estimation [1]. Thus, footprints have more value in forensic identification, and they can be obtained from the scene of a crime and used as evidence for personal identification, such as estimation of stature, sex, race, etc. [15].

Adult stature is usually attained somewhere between the early teens and early 20s. It will be most commonly reached during the mid-teens in females and the late-teens in males. Thus, stature is usually a direct parameter that can be established by measuring footprint dimensions [16]. Dr. Michael Nirenberg, a forensic podiatrist, once stated that a footprint tells you more than a fingerprint. Thus, the current study aims to identify individuals by footprint measurements [17].

Our results indicate that stature can be moderately estimated by footprint dimensions and that sex can also be determined. However, exact estimation may not be possible because there may always be an estimated error. In the current study, the participants were aged between 18 and 23 years. Since all participants were recruited from a single institution, the findings reflect the characteristics of this group and may not represent the wider South Indian population. This age range was chosen because both adult stature and foot size are fully achieved by 18 years. Therefore, only individuals older than 18 years were considered as participants in the study [18].

In the current study, we found a relationship between various footprint dimensions and statures. Our results showed that stature can be successfully estimated using footprint dimensions with ball breadth, showing moderate but statistically significant correlation (r=0.473). This indicates that among all the parameters considered, the footprint width is the most informative predictor of an individual's height in this particular population. Although men and women differed significantly in footprint dimensions, sex-specific correlation/regression models were not developed in the present work and will be considered in future studies.

Our findings are consistent with previous footprint-based stature studies such as those by Kanchan et al. [1], Krishan [8], and Mukhra et al. [12], which also reported moderate correlations between footprint measurements and height in different populations. The moderate correlation values in the present study (r<0.5) may reflect biological variability in foot morphology, minor measurement error, and the relatively narrow stature range within our institutional cohort. Although we used simple correlation analysis to meet the objectives of the study, future research with larger multicentric samples may benefit from applying multivariate regression or discriminant analysis to improve predictive accuracy.

This study showed the presence of sexual dimorphism, which aligns with previous studies indicating that men generally have larger footprint dimensions than women. Furthermore, this study is limited to only young medical students, and only the right foot is compared, which could be a possible limitation. Compared to prior studies, our correlation values are moderate, but our findings are consistent with the literature showing that footprint dimensions, especially foot breadth, can be useful in forensic investigation for stature estimation for individual identification. Overall, this research reinforces the practical utility of footprints and their analysis in individual identification. This information and methodology can be further used for large multiregional datasets to identify victims during mass disasters.

The present study was designed to assess basic associations between footprint dimensions and stature, as well as sexual dimorphism. Therefore, correlation analysis and independent t-tests were chosen as appropriate primary statistical tools. More advanced methods, such as ANOVA or multivariate regression, would require broader sample variability and larger, multicenter datasets and were beyond the scope of the current objectives. These approaches are recommended for future population-based modeling.

Previous studies from North India, Egypt, and Bangladesh have proposed population-specific regression models for stature estimation. However, similar models for South Indian adults are limited, despite known regional and ethnic variations in foot morphology. The present study contributes baseline data for this region and highlights the need for developing robust multivariate regression models tailored to the South Indian population.

## Conclusions

This study demonstrated that all footprint dimensions showed a statistically significant positive correlation with stature, with correlation coefficients ranging from r = 0.34 to 0.47. Among all parameters, ball breadth (BB) exhibited the highest correlation with stature. Significant sexual dimorphism was observed across all footprint dimensions, with male participants consistently exhibiting higher mean values.

The study sample was restricted to students of a single institution, which may limit the generalizability of findings. Only the right foot was analyzed, and sex-specific regression models were not developed. Future studies with larger, multicentric samples and application of multivariate regression or discriminant analysis are recommended to enhance prediction accuracy and establish population-specific reference equations.
